# Re-drawing the Maps for Endemic Mycoses

**DOI:** 10.1007/s11046-020-00431-2

**Published:** 2020-02-10

**Authors:** Nida Ashraf, Ryan C. Kubat, Victoria Poplin, Antoine A. Adenis, David W. Denning, Laura Wright, Orion McCotter, Ilan S. Schwartz, Brendan R. Jackson, Tom Chiller, Nathan C. Bahr

**Affiliations:** 1 Division of Infectious Diseases, Department of Internal Medicine, University of Kansas, Kansas City, KS USA; 2 Department of Internal Medicine, University of Kansas, Kansas City, KS USA; 3 Centre d’Investigation Clinique Antilles-Guyane, Inserm 1424, Centre Hospitalier de Cayenne, Cayenne, French Guiana; 4 Faculty of Biology, Medicine, and Health, University of Manchester, Manchester Academic Health Science Centre, Manchester, UK; 5 Geographic Research Analysis and Services Program, Division of Toxicology and Human Health Services, Centers for Disease Control and Prevention, Atlanta, GA USA; 6 Mycotic Branch, Centers for Disease Control and Prevention, Atlanta, GA USA; 7 Division of Infectious Diseases, Department of Medicine, Faculty of Medicine & Dentistry, University of Alberta, Edmonton, AB Canada

**Keywords:** Histoplasmosis, Coccidioidomycosis, Blastomycosis, Paracoccidioidomycosis, Talaromycosis, Emergomyces, Endemic fungi

## Abstract

Endemic mycoses such as histoplasmosis, coccidioidomycosis, blastomycosis, paracoccidioidomycosis, and talaromycosis are well-known causes of focal and systemic disease within specific geographic areas of known endemicity. However, over the past few decades, there have been increasingly frequent reports of infections due to endemic fungi in areas previously thought to be “non-endemic.” There are numerous potential reasons for this shift such as increased use of immune suppressive medications, improved diagnostic tests, increased disease recognition, and global factors such as migration, increased travel, and climate change. Regardless of the causes, it has become evident that our previous understanding of endemic regions for these fungal diseases needs to evolve. The epidemiology of the newly described *Emergomyces* is incomplete; our understanding of it continues to evolve. This review will focus on the evidence underlying the established areas of endemicity for these mycoses as well as new data and reports from medical literature that support the re-thinking these geographic boundaries. Updating the endemic fungi maps would inform clinical practice and global surveillance of these diseases.

## Introduction

Histoplasmosis, coccidioidomycosis, blastomycosis, paracoccidioidomycosis, talaromycosis, and emergomycosis are termed endemic mycoses because of their historically regular occurrence in limited geographic ranges, and are recognized as substantial causes of morbidity and mortality particularly in the setting of HIV/AIDS, other immunosuppressive medical conditions, or the use of immunosuppressive medications [1–3]. Due to lack of adequate surveillance data worldwide, especially in low-resource settings, it is difficult to estimate the true burden and geographic distribution of these conditions [2]. Further, in some cases, the best diagnostic tests are not widely available outside of the USA (e.g., *Histoplasma* antigen testing), meaning diagnosis requires a high index of suspicion [3, 4]. In recent years, increased attention has been paid to endemic mycoses diagnosed outside of their established geographic ranges, including many with no known exposure to endemic regions. Thus, re-thinking our understanding of the established geographic distribution of these infections is warranted [5–14]. In this review, we will examine the evolving geographic landscape of endemic mycoses worldwide and further underscore the need for improved surveillance, availability of diagnostic tests, and disease awareness among healthcare providers about the wider than previously thought distributions of these diseases.

## Methods

**Fig. 1:**
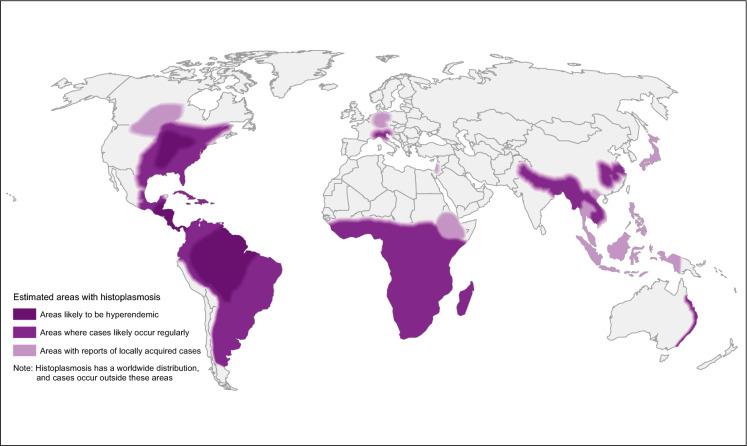
World map estimating regions most likely to have histoplasmosis based on literature review

We reviewed the literature on PubMed using search terms “histoplasmosis,” “coccidioidomycosis,” “blastomycosis,” “talaromycosis,” “penicilliosis,” and “emergomycosis” with the intent of detecting the widest geographic scope of these diseases as possible, although this was not designed to be a systematic review. We searched for case reports, cohort studies, and studies of skin testing, seropositivity, environmental surveys and outbreaks. Where numerous case reports exist, earlier case reports were excluded for purposes of brevity, assuming their inclusion would not add to the overall message (e.g., for a given disease, we included two of 33 case reports from one country). Figure 1 refers to hyperendemic areas of histoplasmosis—these are areas with known high rates of infection and/or skin histoplasmin positivity or seroprevalence. Other categories in this figure are based on frequency of case reports and background prevalence in the context of expert opinion. Only cases without travel to previously known endemic areas were included. For traditionally non-endemic areas where only rare cases have been reported without culture or molecular confirmation, results should be interpreted with caution.

### Histoplasmosis

Histoplasmosis in humans is acquired primarily by inhalation of spores of *Histoplasma capsulatum* var. *capsulatum* or *Histoplasma capsulatum* var. *duboisii* [15]. There is an additional variety, *H. capsulatum* var. *farciminosum*, which has predominantly been described as an equine pathogen but, based on molecular analyses, may have a broader host range which could include humans [16, 17]. Histoplasmosis was first described by Samuel Darling in a worker during construction of the Panama Canal in 1906 [18]. As histoplasmosis was further characterized, it was understood to be intensely endemic in the Ohio and Mississippi River Valleys in the USA as well as in Central and South America [19, 20]. More recently, analysis of phylogenetics and phenotypic characteristics of *Histoplasma* isolates show distinct differences between isolates from different regions suggesting they may actually represent distinct *Histoplasma* species [21]. Within the traditional endemic areas, *Histoplasma* is often found concentrated in areas of “microfoci” which are characteristically areas of soil contaminated with bird or bat guano such as caves, tunnels, chicken coops, or areas of excavated soil [19]. In recent decades, the HIV/AIDS pandemic and the increased use of immunosuppressive agents have led to cases of histoplasmosis reported from previously “non-endemic areas” and have revealed the truly global distribution of histoplasmosis [12, 19] (Table 1).

**Table 1:** Selected areas of *Histoplasma* endemicity outside the North America based on the histoplasmin skin reactivity

Country, year, region	Number tested (population)	Histoplasmin skin test positivity (%), (location if multiple in study)
Africa		
Mali, 1969 [304]	1253 (school children)	6.0
Nigeria, 2018 [50]	735 (HIV-infected patients)	0 (Lagos), 3 (Yola), 2 (Ilorin), 6 (Calabar), 3 (Ibadan), 15 (Benin)
Nigeria, Anambra State, 1996 [51]	40 (cave guides, traders, farmers near a cave), 620 (traders, farmers, palm oil workers)	35.0 8.8
Nigeria, 1991 [305]	1087 (healthy subjects), 226 (pulmonary hospital patients)	1.7-5.0 (*Hcc*), 0.5-4.5 (*Hcd*) 8.9 (*Hcc)*, 6.6 (*Hcd*)
Somalia, Mogadishu and Jilib, 1979 [52]	1014 (NA)	0.3
Uganda, 1970 [53]	1114 (residents)	3.9
Asia		
Bangladesh, 1971 [67]	2572 (pulmonary disease patients)	17.9
China, 2001 [68]	735 (hospitalized patients and healthy residents)	8.9 (Hunan), 15.1 (Jiangsu), 2.1 (Xinjiang)
China, Sichuan Province, 1996 [306]	271 (healthy students and workers) 28 (hospitalized TB patients)	21.8 28.6
India, 1955 [61]	962 (NA)	1.9
India, Delhi, 1962 [61]	8062 (NA)	6.8
India, Kolkata (Calcutta), 1956 [61]	4855 (NA)	0.7
Indonesia, 1956 [75]	2542 (students, hospital patients, nurses)	2.7 (children), 9–12 (adults), Jakarta
Indonesia, 1956 [307]	281 in Surabaya, 340 in Kedisan (school children/villagers)	32 (Surabaya), 63.6 (Kedisan)
Indonesia, Medan, 1997 [308]	1265 medical students	13.6
Malaysia, Sarawak, 1963 [309]	181 school children/hospitalized patients	0.5
Malaysia, Kuala Lumpur, 1964 [310]	224 adults	10.5
Malaysia, Sabah, 1971 [76]	3824 (residents)	11.8
Myanmar, 1952 [96]	3558 (prisoners)	14.5-27.1 (Lower and Rangoon), 4.0–8.4 (Upper) 86.4 (Maguee)
Philippines, Luzon Island, 2001, 1964 [74]	143 (electric company employees)	25.9
Philippines, Manilla, 1964 [311]	2577 (naval recruits)	6.4
Thailand, 1966-1968, [312]	NA (NA)	3–9 (central), 7–14 (northern), 15–36 (southeast and southern)
Thailand, Bangkok, 1967 [313]	497 (medical/nursing students)	5.6
Thailand, 1968 [73]	4211 (prisoners)	14 (northern), 9 (central), 36 (southern)
Vietnam, 1956, Saigon [307]	303 school children/villagers	33.7
Caribbean		
Barbados, 1981 [314]	103 (NA)	4
Trinidad, 1981 [314]	86 (NA)	42
Central and South America		
Argentina, San Martin City, 1996 [315]	315 (children)	9.2
Belize, 1978 [316]	141 (NA)	40
Brazil, Amazon, 1994 [317]	NA (Tupi-Monde Amerindian populations)	78.7 (Surui), 5.8 (Gaviao), 80.5 (Zoro)
Brazil, Recife, 1966 [318]	1006 (hospital patients)	20.5
Brazil, Belem, 1966 [318]	258 (hospital patients and medical students)	43.4
Brazil, Minas Gerais State, 1996 [319]	417 (miners)	17.5
Colombia, 1968[320]	NA (NA)	21.0
Guatemala, 1960 [321]	821 (hospital patients)	23–81
Mexico, Guerrero State, 1997 [322]	139 (cave guides, guano collectors, fishermen)	87.3 (Jutlahuaca), 76.9 (Olinala), 3.8 (Coyuca)
Venezuela, Bolivar State, 2004 [323]	157 (residents, farmers)	42.7
Europe		
Italy, Po Valley, 1994 [109]	776 (students)	1.2

*Hcc—Histoplasma capsulatum* var. *capsulatum*; *Hcd*—*Histoplasma capsulatum* var. *duboisii*; NA—information not available

Within the USA, while the Mississippi and Ohio River Valley regions are highly endemic, histoplasmosis occurs well-beyond these boundaries. In a study of histoplasmosis cases among the US Medicare beneficiaries, nearly 12% of cases were from non-endemic areas [14]. A study of histoplasmosis diagnoses among privately insured patients showed that 20% of cases occurred outside traditionally defined endemic regions [5]. While these larger studies were unable to evaluate cases on an individual basis for travel to endemic areas, other publications report several human cases of autochthonous histoplasmosis from areas not previously thought to be endemic. These include California, Arizona, Idaho, Montana, and New York as well as states north of the classical endemic area such as Minnesota, Wisconsin, and Michigan [6, 22–25]. Moreover, veterinary cases have been reported to extend into southwest states such as New Mexico and Colorado and as far north as Alaska [26, 27]. In Canada, histoplasmosis is endemic in Quebec and Ontario along the St. Lawrence Seaway and the Great Lakes Drainage Basin [7, 28, 29]. More recently, several laboratory confirmed cases with local acquisition have been reported in Alberta, and there has been at least one confirmed common source outbreak in Saskatchewan (IS Schwartz, unpublished data) [30, 31].

Histoplasmosis is endemic throughout much of Central and South America with an estimated 32% histoplasmin skin test positivity throughout Latin America (with regional variability) [32]. Chile has an estimated prevalence of 0.1% and, in a 2017 case series, all nine cases occurred in the setting of foreign travel or immigration [32, 33]. In Argentina, 30–40% of the population has been estimated to have had exposure to histoplasmosis [34]. In Mexico, an estimated 112–325 cases of acute pulmonary or disseminated histoplasmosis have been reported annually, primarily in the central and southeastern states of Veracruz, Oaxaca, Campeche, Tabasco, and Chiapas, although this is considered a significant underestimation due to variable diagnostic methodologies, lack of surveillance programs, and lack of diagnostic capabilities in many areas [34, 35]. In Central America, reported histoplasmin skin test positivity ranges from 37% in Costa Rica and Nicaragua to 57% in Guatemala [32]. In South America, disseminated histoplasmosis is increasingly identified in persons with new HIV diagnoses and is estimated to be as common in this setting as tuberculosis [32, 36–39]. Further, the true incidence of histoplasmosis in HIV/AIDS is likely substantially higher than currently recognized due to limited availability of *Histoplasma* antigen testing. In Brazil, the introduction of such testing led to a 53.8% increase in diagnostic yield [40]. Scattered cases of histoplasmosis have been identified throughout the Caribbean islands including outbreaks in the Dominican Republic and Cuba with endemicity recognized in Jamaica [41–44]. Histoplasmin skin test positivity rates as high as 42% in Trinidad and Tobago suggest that Caribbean cases may be under-recognized as well [45].

Histoplasmosis in Africa is caused by both *H. capsulatum* var. *capsulatum*, which is found throughout much of Africa, and *H. capsulatum* var. *duboisii*, which has been reported throughout West Africa (with the majority of cases from Nigeria), the Democratic Republic of the Congo, Uganda, Tanzania, and scattered throughout central and eastern Africa including isolated cases from Madagascar [46–48]. In contrast to *H. capsulatum* var. *capsulatum*, *H. capsulatum* var. *duboisii* predominantly causes skin and soft tissue infections and rarely involves the lungs [46, 49]. A literature review of all published cases of histoplasmosis from Africa found a total of 470 cases from 1972–2017, with the highest number of cases originating from West Africa [46]. The majority of West African cases are reported from Nigeria, where studies evaluating histoplasmin skin sensitivity have shown rates ranging from 4.4% in a predominantly urban population up to 35% near a bat cave in a rural part of the country [46, 50, 51]. Additional studies of histoplasmin sensitivity in Uganda showed a positivity rate ranging from 0.4%–10% in separate Ugandan districts, while a study in Somalia found a total positivity rate of only 0.3% [52, 53]. More recently, *Histoplasma* antigen and anti–*Histoplasma* antibody studies have been performed, with zero of 100 Somali refugees residing in Kenya exhibiting seropositivity for anti-*Histoplasma* IgG and 1.3% of Ugandan persons living with HIV/AIDS exhibiting anti-*Histoplasma* IgG seropositivity (with no positive anti-*Histoplasma* IgM or *Histoplasma* serum, urine, or cerebrospinal fluid antigens among 151 subjects) [54, 55]. One striking study from Maputo, Mozambique, found that 58% of HIV-infected patients hospitalized with respiratory infections or Kaposi’s sarcoma were diagnosed with histoplasmosis via nested PCR [56]. The majority of reported histoplasmosis cases with HIV coinfection have been caused by *H. capsulatum* var. *capsulatum*; however, *H. capsulatum* var. *duboisii* is being increasingly recognized in HIV coinfected patients and has been shown to cause disseminated disease in this population [46, 49, 57–59].

Within Asia, *Histoplasma* has been known to be present in certain areas for many years [60]. *Histoplasma* was first isolated from soil in Malaysia in 1963, and Randhawa reviewed 30 possible autochthonous cases from India, Malaysia, Indonesia, Singapore, Thailand, Vietnam, and Japan in 1970 [61, 62]. There have been 144 cases of histoplasmosis recorded from 1954 through 2017 in India with the majority of reports from West Bengal, Assam, Bihar, Delhi, Haryana, Punjab, and Uttar Pradesh; as in many other settings, histoplasmosis is felt to be underdiagnosed in this country [63–65]. Histoplasmin sensitivities in Kolkata and Delhi range from 4.7–12.3% [61]. Histoplasmin sensitivity in Bangladesh was found to be 17.9% with 16 reported cases of histoplasmosis in the medical literature from 1982 to 2013 [66, 67]. One study found histoplasmin positivity in China of 9.0% overall with higher values in Hunan and Jiangsu provinces [68]. A review of 300 cases of histoplasmosis in China from 1990–2011 (257 disseminated, 22% HIV infected) found that 75% of the cases were from regions along the Yangtze River in southeastern China, with all but 17 cases thought to be autochthonous [69]. Moreover, the use of bat guano as an herbal medicine may increase the risk of acquiring histoplasmosis in endemic areas [70]. In Japan, histoplasmin sensitivity is negligible (except in those exposed to imported soils) and local bat guano does not contain *Histoplasma* [61, 71]. The majority of cases of histoplasmosis in Southeast Asia have been reported from Thailand, where 1253 cases of disseminated histoplasmosis among HIV-infected persons were reported to the Ministry of Public Health from 1984 to 2010 [72]. Histoplasmin sensitivity in Thailand is as high as 34.4% in south and central Thailand and as low as 4.8% in north and northeast Thailand, although it is hypothesized that this may be an over-estimation of true exposure due to cross-reactivity with *Talaromyces marneffei* antigen, which is also present in the region [54, 73]. A study of Burmese and Hmong refugees residing in Thailand found only 2/199 with seropositivity for anti-*Histoplasma* IgG [54]. Histoplasmin sensitivity in adults ranged from 9 to 12% in Indonesia and Malaysia and as high as 26% in the Philippines with cases reported in each of these countries [74–79]. Additional data suggest histoplasmin positivity of greater than 50% in parts of Myanmar and additional cases of histoplasmosis from Laos, Cambodia, Vietnam, Indonesia, Malaysia, Myanmar, the Philippines, and Singapore [60, 80–96]. Scattered cases of autochthonous histoplasmosis have been reported in Australia dating back to 1948, and *H. capsulatum* has been isolated from fowl yards and caves within the country [97, 98]. An analysis of 63 proven histoplasmosis cases deemed 41 to have been acquired locally, primarily in Queensland and New South Wales which have large areas of tropical and subtropical environments [99]. Recently, the first Middle Eastern autochthonous case was diagnosed in Israel [100].

In Europe, histoplasmosis is predominantly an imported disease [101]. In a review of 118 proven or probable histoplasmosis cases in Europe over a five-year period, Ashbee and colleagues found that all but eight cases had a history of travel to or migration from an endemic area [102]. The majority of European autochthonous cases of histoplasmosis have been identified in Italy, and *H. capsulatum* has been isolated from soil in the Po River valley where higher histoplasmin skin test positivity rates of 1.2% occur [103–109]. Ashbee’s review also identified one case from Germany, where histoplasmosis has been diagnosed in badgers and hedgehogs [110, 111], and Turkey, where two other presumed autochthonous cases have been published [112, 113].

Our understanding of the distribution has improved such that rather than seeing histoplasmosis as a disease of the Central USA and parts of Central and South America, the map shown in Fig. 1 better reflects our current understanding of histoplasmosis endemicity. Histoplasmosis is truly endemic in much of the world. Further, in most settings, histoplasmosis is presumed to be underdiagnosed due to lack of available diagnostic tests and/or clinician awareness, or due to misdiagnosis.

### Coccidioidomycosis

Coccidioidomycosis (Valley Fever) is caused by two epidemiologically and genetically diverse species, *C. immitis* and *C. posadasii* [34, 114–120]. *C. immitis* was first discovered in 1892 in Buenos Aires and misidentified as a protozoan; years later, it was correctly identified as a fungus [34, 121–124]. In 1957, Edwards et al. used skin testing to delineate the endemic areas within the USA [6, 121]. Subsequently, public health surveillance as well as various serological and molecular methods have been utilized to help improve understanding of the geographic distribution of coccidioidomycosis within the USA [119, 125–127]. In 2002, Fisher et al. isolated two distinct pathogenic species based on phylogenetic analyses: *C. immitis* and *C. posadasii* [128]. *C. immitis* is primarily found in the Central Valley of California but has now been found as far north as eastern Washington state [119, 129–134]. *C. posadasii,* is found in the desert areas of Arizona, Texas, Utah, Mexico, and Central and South America [6, 133, 135–140]. However, there is geographic overlap between the two species in Southern California [122].

Southern Arizona and the San Joaquin Valley region in California have long been identified as hyperendemic areas, and these states account for 95% of all reported cases of coccidioidomycosis within the USA [141–144]. Although most cases are not associated with outbreaks, they typically involve disruption of the soil, including military maneuvers, construction work, earthquakes, landslides, and armadillo-hunting expeditions [145–149]. Some of the highest rates of coccidioidomycosis have occurred in people incarcerated in some of the prisons in California’s Central Valley, and health officials have implemented policies to reduce risk and severe disease in these populations [143, 150, 151]. Interestingly, the incidence of coccidioidomycosis seems to be rising nationally during the last couple of decades in both endemic and non-endemic regions, and a total of 95,317 cases were reported between 2011 and 2017; while the incidence decreased in Arizona to 101 per 100,000 persons in 2017 from 261 in 2011, the incidence increased in California to 18.2 from 15.7 during the same time period [152]. The Centers for Disease Control and Prevention (CDC) reported a 58% increase in coccidioidomycosis incidence in Arizona from October 2017 to March 2018 compared to the preceding years, and California saw the highest year on record in 2017. Turabelidze et al. reported a fivefold increase in the incidence of cases in Missouri from 0.05 per 100,000 population in 2004 to 0.28 per 100,000 in 2013, with about a quarter of the cases either having no reported travel to the known endemic areas or no travel history [139, 153]. Meanwhile, a surveillance study in 14 states by Benedict et al. in 2016 identified Utah, Nevada, and New Mexico as low endemic areas compared to 11 other states including Missouri that were deemed non-endemic [135]. The study also highlighted the need for increased awareness of coccidioidomycoses in areas of low endemicity to avoid delay in its accurate diagnosis [135].

While coccidioidomycosis is a reportable disease within 26 states plus the District of Columbia as of February 2019 (Table 2), it is not a notifiable disease in the other 24 US states or in Latin America (with the exception of Argentina) [132, 154, 155]. In 1944, Gonzales-Ochoa was the first to conduct skin testing in Sonora and Baja California demonstrating 16% reactivity rates [156]. In 1961, three endemic zones were recognized in a systematic study in Mexico: Northern zone (bordering the USA and including Baja California, Sonora, Chihuahua, Coahuila, Nuevo Leon, and Tamaulipas); Pacific Littoral Zone (extending southeast from the Northern Zone to Michoacán), and the Central zone (extending from the northeastern border of Coahuila and ending at the Michoacán border) [156]. Skin testing has thus been used to establish the endemic areas within Mexico with the highest proportion of reactors in the states of Baja California, Sonora, Sinaloa, Nuevo León, Coahuila, Tamaulipas and Chihuahua in the northwest [156, 157]. However, with lack of reporting and diagnostic capability, very little is known about current endemic areas. This translates to a dearth of knowledge regarding the endemic areas within the region and a reliance on published cases [34, 35, 145, 157, 158].

**Table 2:** States in which selected endemic mycoses are notifiable as of February 2019

States	Histoplasmosis	Coccidioidomycosis	Blastomycosis
Alabama		✓	
Arizona	✓	✓	✓
Arkansas		✓	
California	✓	✓	
Delaware		✓	
District of Columbia		✓	
Illinois	✓		
Indiana	✓	✓	
Kansas	✓	✓	
Kentucky	✓		
Louisiana	✓	✓	✓
Maryland		✓	
Michigan	✓	✓	✓
Minnesota	✓	✓	✓
Missouri		✓	
Montana		✓	
Nebraska		✓	
Nevada		✓	
New Hampshire		✓	
New Mexico		✓	
North Dakota		✓	
Ohio		✓	
Oregon		✓	
Pennsylvania	✓		
Rhode Island		✓	
South Dakota		✓	
Utah		✓	
Washington		✓	
Wisconsin	✓	✓	✓
Wyoming		✓	

Content source: Centers for Disease Control and Prevention, National Center for Emerging and Zoonotic Infectious Diseases (NCEZID), Division of Foodborne, Waterborne, and Environmental Diseases (DFWED)

**Fig. 2:**
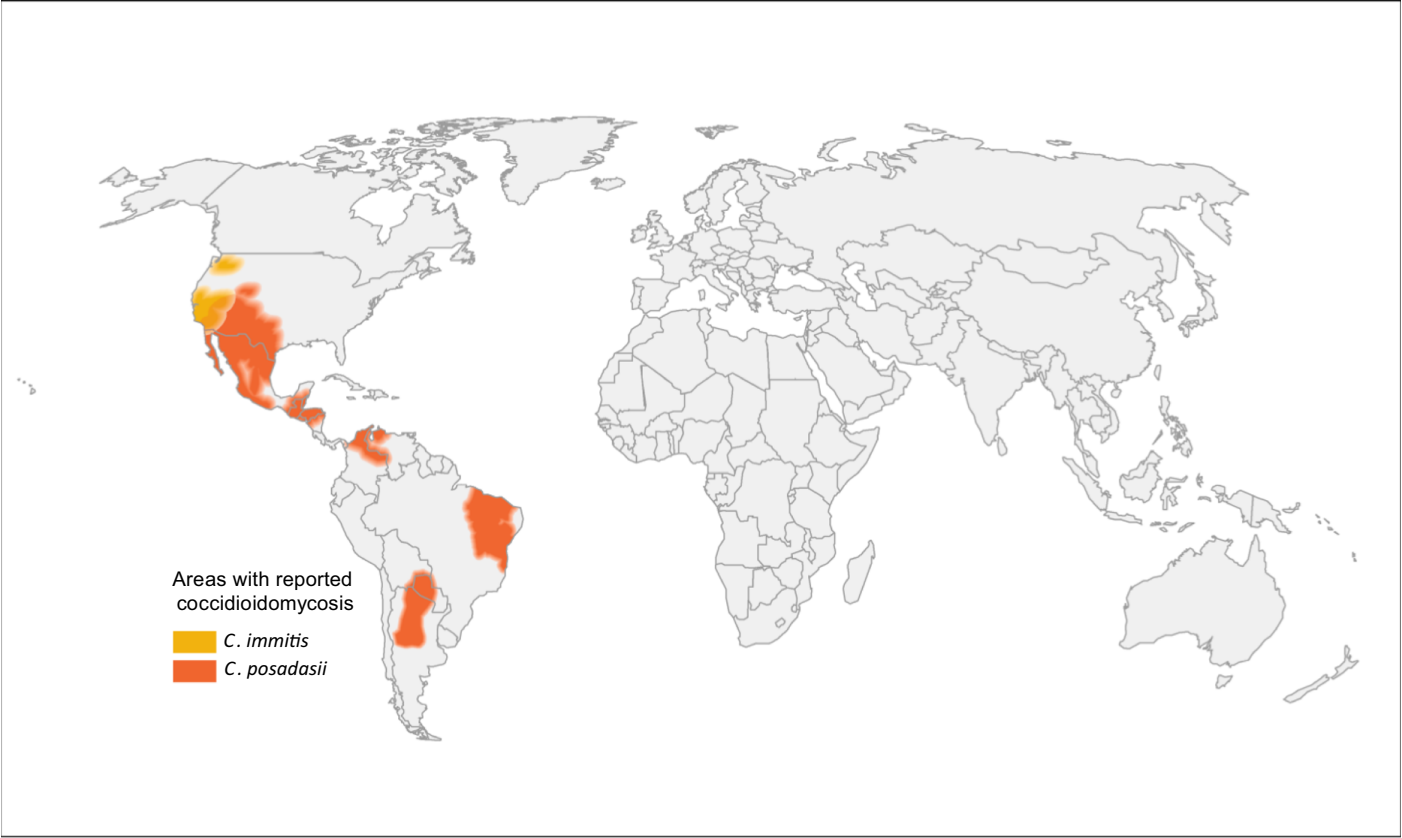
World map estimating regions with coccidioidomycosis based on literature review

Within South America, numerous geographically isolated areas of endemicity have been discovered including the northeastern areas of Colombia; Zulia, Lara, and Falcon states in Venezuela; the Chaco region in Argentina and Paraguay including the provinces of Catamarca, La Rioja, and San Luis; and the Piaui, Maranhao, Ceara, and Bahai states of Brazil [123, 155, 157]. In Central America, skin testing was first conducted by Andrade in 1945, reporting a low prevalence of reactors in Guatemala [159]. In 1953, Trejos et al. reported the first case in a resident of Honduras, and conducted skin testing the same year to establish endemicity in the Comayagua Valley of Honduras [160]. Since then, areas of endemicity have been identified in the Montague Valley of Guatemala and the Comayagua Valley of Honduras based on case reports [161]. Laniado-Laborín et al. and Negroni et al. also propose endemicity in Bolivia [121, 162, 163]. Figure 2 describes the geographic distribution of *C. immitis* and *C. posadasii* worldwide.

### Blastomycosis

**Fig. 3:**
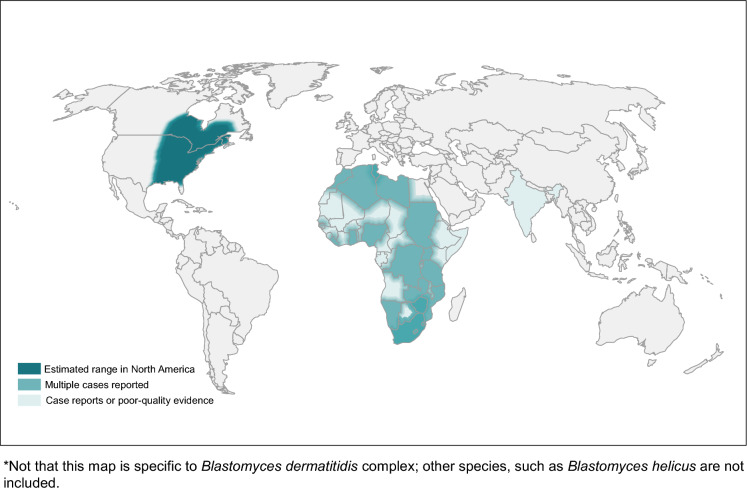
World map estimating regions most likely to have blastomycosis* based on literature review

*Blastomyces dermatitidis*, including the more recently described cryptic species *B. gilchristi* (together referred to herein as *B. dermatitidis* species complex), and *B. helicus* cause disease in humans and animals via inhalation of airborne spores [6, 14, 164–171]. The mycelial form of the fungus primarily dwells in wooded land with damp soil near lakes, waterways and rivers [164, 167, 171–175]. Excavation and construction in endemic areas have been identified as risk factors for disease acquisition [176]. *B. dermatitidis* species complex is endemic in the mid-west, southeast, east and south-central USA; northwest Ontario, Quebec, Manitoba and Saskatchewan in Canada; central, eastern and southern Africa; and India [6, 7, 28, 164, 166, 167, 177–185]. Figure 3 shows the geographic distribution of *B. dermatitidis* species complex worldwide.

Much of the epidemiological data for blastomycosis have been obtained from reports in North America, with fewer reports from Africa and the Asia–Pacific region [181–184, 186–191]. As of February 2019, blastomycosis is only reportable in five states within the USA (Table 2), and while it used to be reportable in Ontario, Canada that has not been the case since 1989 [192]. The lack of mandatory public reporting coupled with the paucity of reliable tests for prior exposure has hindered the epidemiologic understanding of blastomycosis, unlike coccidioidomycosis and histoplasmosis [7, 166, 167, 171, 193–197].

Within the USA, Mississippi has historically had the highest prevalence, while incidence of hospitalizations involving blastomycosis was the highest in Arkansas, Illinois, Kentucky, Tennessee, and Wisconsin [170, 198, 199]. Incidence rates in the hyperendemic areas surrounding Mississippi and Ohio River Valleys vary from 0.5–100/100,000 [171]. Thus far, blastomycosis has been reported in Alabama, Arkansas, Colorado, Georgia, Illinois, Indiana, Iowa, Kentucky, Louisiana, Michigan, Minnesota, Mississippi, Missouri, Nebraska, New York, North Carolina, North Dakota, Ohio, Pennsylvania, South Carolina, South Dakota, Tennessee, Texas, Vermont, West Virginia, and Wisconsin [14, 165, 170, 175, 180, 193, 194, 199–211]. A number of these states clearly fall outside of the typically described endemic area, and it is unclear whether they truly belong to *B. dermatitidis* species complex or *B. helicus* (Fig. 3). Within Canada, blastomycosis has been reported in the provinces adjoining the Great Lakes including Manitoba, Ontario and Quebec [7, 179, 212–214]. More recently, Lohrenz et al. reported 15 cases of blastomycosis in southern Saskatchewan of which nine had never been to a known endemic region [215]. The endemicity of *B. dermatitidis* in Asia–Pacific region remains controversial [175, 177]. In India, the organism was first isolated from a bat in Delhi in 1982 and human case reports with pulmonary and cerebral lesions have been described; however, Savio and colleagues subsequently noted that previously reported cases had prior travel to an endemic area in the USA or poor quality of evidence confirming the disease [175, 177, 187–190, 216]. In Africa, *B. dermatitidis* has been primarily reported in Tunisia, South Africa and Zimbabwe although cases have been reported in individuals from Algeria, Libya, Sudan, Morocco, Gambia, Namibia, Mozambique, Zambia, Tanzania, Uganda, Rwanda, the Democratic Republic of Congo, Nigeria, Liberia, and Ghana; while majority of these cases have disease confirmation based on cultures or histopathology, the quality of evidence is poor for others [181–184, 191, 217–228].

Less is known about the distributions of *B. helicus. B. helicus* has been reported in western regions of North America including Alberta and Saskatchewan, Canada, as well as Colorado, Idaho, Montana, Nebraska, Northern California, Texas, and Utah in the USA [229].

### Paracoccidioidomycosis

Paracoccidioidomycosis is a systemic mycosis caused by *Paracoccidioides brasiliensis* and *Paracoccidioides lutzii* [230–241]. Paracoccidioidomycosis is a rare disease worldwide, but is a frequent AIDS-defining opportunistic infection in Latin America, and is now recognized as a neglected tropical disease by the World Health Organization [230, 231, 235, 239, 242–251]. *P. brasiliensis* is endemic in large parts of South America, with the greatest prevalence in southeast, south, and central-west Brazil; Venezuela and Columbia, followed by northern Argentina, eastern Paraguay, and the Cuenca River valley in Ecuador [155, 230, 232, 235, 238, 239, 245, 246, 252–257]. Southern Mexico and Central America have lower rates but are also endemic [238, 258]. Because these data are inferred from case reports and retrospective studies of hospitalized patients, incidence rates are postulated to be higher [34, 155, 238, 239, 248, 253, 257]. Furthermore, climate change, human migration, the expansion of agricultural activities, and highway construction have affected the epidemiology of *Paracoccidioides*, which is now expanding from the south and southeast to the central-west and north regions of Brazil [238, 249, 259]. All cases of *P. brasiliensis* reported outside of endemic regions were acquired via travel to endemic areas [101, 238, 256, 260–263] (Table 3). *P. lutzii* was only recently identified as a new species by multi-locus sequencing studies, and is known to be endemic in central, mid-west, and northern Brazil, Ecuador, and Venezuela [34, 236, 238, 241, 264].

**Table 3:** Areas of endemicity for *Paracoccidioides* species

Region/Species	Basis of endemicity
*P. brasiliensis*	
South America	
*Brazil*	
Sao Paulo [253, 254, 257, 324–326] Espírito Santo [233, 253] Rio de Janeiro [253, 327, 328] Minas Gerais [252–254, 257, 329] Rondônia [232, 257] Mato Grosso [257] Bahia [253, 257] Mato Grosso do Sul [257, 330] Paraná [257, 331, 332] Rio Grande do Sul [333, 334] *Argentina* Corrientes [240] Formosa [335] *Venezuela:* San Felix city, Bolivar [336]	Multiple reports ranging from 1–1219 cases during 1960–2012 Two studies reporting 83–444 cases during 1978–2012 Multiple reports during 1978–2012 ranging from 3–36 cases Multiple reports during 1978–2009 ranging from 50–252 cases Two reports during 1988–2012, 3 and 2163 cases Two reported cases (1988–1996); confirmation based on serology or histopathology Two reports during 1978–2012 of 1 and 30 cases One human case and 280 cases in cattle. Multiple reports ranging from 1–102 human cases Two reports of 61-123 human cases during 1966-2009 Endemicity based on positive skin tests in 52/455 humans in one study One case series of 22 human cases Endemicity based on positive skin test in 28/275 humans
North America	
*Mexico* Gulf of Mexico [258] Pacific littoral [258]	51 human cases reported during 1972–2012 18 human cases reported during 1972–2012
*P. lutzii*	
South America *Brazil,* Para [255]	Two human cases; confirmation based on phylogenetic analysis
*Unspecified Paracoccidioides species*	
South America	
*Brazil*	
Amazonas [337] Para (284) Acre [337] Rondonia [337] Federal Territory of Roraima [337] Maranhao [338] Rio de Janeiro [249] Minas Gerais [241] Bolivia [231]	NA NA NA NA NA Twenty-nine reported human cases during 2004–2010 Outbreak with report of eight human cases 2015–2016 One human case report, confirmed on histopathology One human case report; article is in Japanese, and information regarding diagnosis is not available.

*NA* information not available

### Talaromycosis

Talaromycosis is a common, AIDS-defining opportunistic infection in South and Southeast Asia [265–273]. *Talaromyces marneffei* (formerly *Penicillium marneffei*) is a soil dwelling fungus that causes disease in humans via inhalation or inoculation of conidia [270]. Di Salvo et al. described the first naturally acquired infection in a patient with Hodgkin’s lymphoma in 1973 (the patient was in the USA but had been to Southeast Asia three years prior), and only a handful of cases were reported in Thailand until 1984 [274, 275]. Subsequently, alarmingly high incidence rates were observed in Southeast Asia in 1988, paralleling the HIV-AIDS epidemic [270].

*T. marneffei* is endemic in southwest China (particularly Guangxi province) but seems to be increasing in much of mainland China with 668 cases reported between 1984 and 2009 [268–270, 273, 276, 277]. Thailand, Hong Kong, northeastern India (particularly Assam and Manipur states), Taiwan, Laos, Cambodia, Malaysia, Myanmar, Indonesia and Vietnam are other endemic areas based on autochthonous case reports [266, 268–271, 273, 278–287] (Table 4). In China, 8% of *T. marneffei* cases occur in healthy individuals; additionally, talaromycosis is an important presentation of adult-onset immunodeficiency syndrome, which is more common in Southeast Asia [288]. Interestingly, case reports of talaromycosis in Togo and Ghana, in West Africa, have occurred without known travel to endemic regions [273].

**Table 4:** Areas of *Talaromyces marneffei* endemicity

Region	Basis of endemicity
Republic of China	
Guangxi [272, 277, 339] Other provinces [276, 340–342]	Multiple reports ranging from 8–109 human cases Multiple reports ranging from 1–668 human cases, 1984-2017
Taiwan [282, 343–345]	Multiple reports ranging from 1–35 cases
Hong Kong [346–353]	Multiple reports ranging from 1–47 cases
Thailand	
Chiang Mai [268, 354] Chiang Ray [355] Khon Kaen [356] NR [357]	Multiple reports in HIV-infected patients ranging from 80–1843 cases during 1990–2004 One case report in an Italian man based on microbiologic confirmation 10.6% of fungal isolates collected from patients with invasive fungal infections during 2006–2011 were *Talaromyces marneffei* One case report in a traveler in Greenland and Denmark from Thailand based on microbiologic confirmation
Vietnam	
Ho Chi Minh City [287, 358–360] Tay Ninh [287] Dong Nai [287] Kon Tum [287]	Multiple reports ranging from 1–719 cases One case report based on microbiologic confirmation One case report based on microbiologic confirmation One case report based on microbiologic confirmation
India	
Manipur [271, 280, 361]	Multiple reports ranging from 1–46 cases
Laos [283, 362]	Two reported cases based on microbiologic confirmation
Myanmar [286]	One case report based on microbiologic confirmation

*NR* not reported

### Emergomycosis

Emergomycosis is a disease caused by infection with thermally dimorphic fungi in the recently described genus *Emergomyces*. The earliest member of the genus, *Es. pasteurianus*, was originally described in 1998 in the genus *Emmonsia* based on genetic and phenotypic similarities to *Emmonsia parva* (since reclassified as *Blastomyces parvus*) and *Ea. crescens*. It remained the outlier in the genus because, unlike *Ea. parva* and *Ea. crescens*, the thermodependant tissue phase was characterized by small, narrow budding yeasts in contrast to large, non-replicating adiaspores. The relevance and taxonomic placement of *Ea. pasteuriana*, as it was then known, was uncertain until 15 years later, with publication of a report of South African patients with advanced HIV disease who developed disseminated disease caused by a novel fungus [289]. Those isolates were closely related to *Ea. pasteuriana*, and eventually prompted a re-examination of global fungal collections for atypical *Emmonsia*-like isolates. What ensued was a taxonomic overhaul of the Ajellomycetaceae, including *Emmonsia* and *Blastomyces*, and the creation of a new genus, *Emergomyces* [290, 291].

There are currently five species of *Emergomyces* (Table 5). *Es. africanus* has been implicated in over 80 cases in South Africa, where it is the most frequently diagnosed endemic mycosis [292, 293]. *Es. pasteurianus* has been described in Italy, Spain, the Netherlands, France, India, China, South Africa, and Uganda. Although two cases from the Netherlands were associated with travel to Morocco and Iraq, other European cases had no significant travel history reported [294–299]. *Es. canadensis* has been described in Saskatchewan, Canada, and in Colorado and New Mexico in the USA. Only one case of infection due to *Es. orientalis* has been described, in China [299–301]. *Es. europaeus* was reported to cause infection just once, when it was isolated from the lung of a German patient on chronic corticosteroids [290, 302].

**Table 5:** *Emergomyces* species by report locations

Species	Case report locations
*Es. africanus* [292, 293]	South Africa
*Es. pasteurianus* [294–299*]*	Italy, Spain, the Netherlands, France, India, China, South Africa, and Uganda
*Es. canadensis* [299, 300]	Canada, USA
*Es. orientalis* [301]	China
*Es. europaeus* [290, 302]	Germany

## Conclusions

Endemic mycoses cause significant morbidity and mortality in immunocompetent and immunocompromised individuals worldwide and each has its own evolving regions of endemicity. Diagnosis is often missed or delayed, especially outside the areas of endemicity, due to a lack of awareness of the pathogen which is due at least in part to a scarcity of data on its geographic distribution [7].

This review summarizes the recent shifts and expansions observed in the prevalence of some of the endemic fungi worldwide. We hypothesize that these changes result from human migration, agricultural practices, occupational exposures, deforestation, soil movement, and climate change [238]. Ongoing disease surveillance is essential to understand these diseases, and wider public health reporting could help detect locally acquired cases and track changes in spatial and temporal distribution. Closer observation would allow for better understanding of the epidemiology of these fungi and improve clinical awareness. Ongoing environmental and epidemiological studies are warranted to accurately estimate the incidences and geographic distribution of these fungi worldwide.

In 1971 Ajello wrote, “Information on the prevalence and incidence of histoplasmosis is extensive when compared with that available for the other mycoses. Much remains to be learned, however, before we have the full picture of its impact on the welfare of human beings” [303]. Truly, despite many reports, the full picture of the impact of histoplasmosis on the welfare of humans remains unclear, in part, because the condition is underfunded and neglected. The same is true to a far greater degree for other endemic fungi.
